# Human pharyngeal microbiota in age-related macular degeneration

**DOI:** 10.1371/journal.pone.0201768

**Published:** 2018-08-08

**Authors:** Eliza Xin Pei Ho, Chui Ming Gemmy Cheung, Shuzhen Sim, Collins Wenhan Chu, Andreas Wilm, Clarabelle Bitong Lin, Ranjana Mathur, Doric Wong, Choi Mun Chan, Mayuri Bhagarva, Augustinus Laude, Tock Han Lim, Tien Yin Wong, Ching Yu Cheng, Sonia Davila, Martin Hibberd

**Affiliations:** 1 Genome Institute of Singapore,Singapore, Singapore; 2 Singapore Eye Research Institute, Singapore National Eye Center, Duke-NUS Medical School, National University of Singapore, Singapore, Singapore; 3 Department of Ophthalmology, National University of Singapore and National University Health System, Singapore, Singapore; 4 National Healthcare Group Eye Institute, Tan Tock Seng Hospital, Singapore, Singapore; 5 Faculty of Infectious and Tropical Diseases, London School of Hygiene and Tropical Medicine, London, United Kingdom; University of Manchester, UNITED KINGDOM

## Abstract

**Background:**

While the aetiology of age-related macular degeneration (AMD)—a major blinding disease—remains unknown, the disease is strongly associated with variants in the complement factor H (CFH) gene. CFH variants also confer susceptibility to invasive infection with several bacterial colonizers of the nasopharyngeal mucosa. This shared susceptibility locus implicates complement deregulation as a common disease mechanism, and suggests the possibility that microbial interactions with host complement may trigger AMD. In this study, we address this possibility by testing the hypothesis that AMD is associated with specific microbial colonization of the human nasopharynx.

**Results:**

High-throughput Illumina sequencing of the V3-V6 region of the microbial 16S ribosomal RNA gene was used to comprehensively and accurately describe the human pharyngeal microbiome, at genus level, in 245 AMD patients and 386 controls. Based on mean and differential microbial abundance analyses, we determined an overview of the pharyngeal microbiota, as well as candidate genera (*Prevotella* and *Gemella*) suggesting an association towards AMD health and disease conditions.

**Conclusions:**

Utilizing an extensive study population from Singapore, our results provided an accurate description of the pharyngeal microbiota profiles in AMD health and disease conditions. Through identification of candidate genera that are different between conditions, we provide preliminary evidence for the existence of microbial triggers for AMD.

Ethical approval for this study was obtained through the Singapore Health Clinical Institutional Review Board, reference numbers R799/63/2010 and 2010/585/A.

## Introduction

The human throat harbors a complex bacterial community located at the intersection of the digestive and respiratory tracts. This pharyngeal microbiome is dominated by the phyla Firmicutes and Bacteroidetes [1] and is thought to protect against respiratory tract infections and invasive disease by preventing the outgrowth of potentially pathogenic bacteria (reviewed in [2]).

The human complement system is a major innate immune defense against meningococcal disease. Briefly, the binding of complement proteins to antigen-antibody complexes (classical pathway) or to the pathogen surface (alternative pathway) activates a triggered-enzyme cascade, resulting in an amplified response that brings about pathogen lysis, pathogen opsonization, and the recruitment and activation of phagocytes.

To prevent damage to host cells, this potent response is kept under regulatory control. This host cell protection mechanism nonetheless can be exploited by microbes developing complement evasion mechanisms that aid in their pathogenicity [3]. These pathogens include *Neisseria meningitidis* and *Streptococcus pneumoniae*, which are carried asymptomatically in the nasopharynx [4,5]. These microbes exploit this dampening mechanism by factor H sequestration, via binding to domains 6–7 or through carboxyl-terminal domains 19–20 on factor H [5]. The complement factor H (*CFH*) gene encodes a negative regulator of the alternative complement pathway. Individuals homozygous for risk variants of *CFH*, when exposed to *Chlamydia pneumoniae*, were found to face an increased risk of age-related macular degeneration (AMD) progression [6]. Similarly, variants in *CFH* were found to show strong associations with susceptibility to meningococcal disease [7].

Intriguingly, the *CFH* gene is also associated with susceptibility to AMD [8–9], a blinding disease that is an important cause of vision loss in the elderly. It is thought that the disease will afflict 288 million people globally by 2040 [10].

Early-stage AMD, which presents minimal symptoms and is usually detected through routine eye examinations, is characterized by the presence of drusen deposits within the ocular sub-retinal space. Most vision loss occurs during advanced stage disease, which takes two main forms: geographic atrophy ("dry" AMD), characterized by degeneration of large, confluent regions of the retinal pigmented epithelium, and neovascular ("wet") AMD, characterized by invasion of the retina by leaky choroidal blood vessels and macrophages (reviewed in [11]).

Despite extensive research, the etiology of AMD is unknown. It is well known, however, that *CFH* risk variants encode proteins that bind with lower affinity to Bruch's membrane (the multilayered extracellular matrix separating the retina from choroidal blood vessels) and to other complement pathway components, potentially resulting in an impaired inhibitory effect [12–14]. In addition, increased alternative pathway activity has been reported for several AMD-associated risk variants in other complement pathway components and regulators, with protective variants having the opposite effect [15]. Thus, one hypothesis for AMD etiopathogenesis is complement over-activation in response to injury and debris in the retina, rendering increased exposure to complement-mediated damage to host cells. There are postulations that interactions between microbial colonizers and host complement could provide a trigger for AMD [6,16].

In this study, we used 16S rRNA sequencing to comprehensively characterize the pharyngeal microbiomes of 260 AMD cases and 386 controls, thus allowing us to investigate possible roles for specific microbial genera in the pathogenesis of AMD.

## Materials and methods

### Study population and sample collection

Following protocols described previously, we recruited 311 AMD cases at three clinical sites: Singapore National Eye Centre, Tan Tock Seng Hospital and National University Hospital [17]. Ethical Approval was obtained through the Singapore Health Clinical Institutional Review Board (R799/63/2010; 2010/585/A), with written informed consent from participants. AMD diagnosis was made according to standard definitions based on ophthalmic examinations, including dilated fundus photography, fluorescein angiography, indocyanine green angiography and optical coherence tomography. Grading of fluorescein angiograms for presence of choroidal neovascularization was done using a modification from the Macular Photocoagulation Study [18]. Indocyanine green angiography was done to diagnose definitive polypoidal choroidal vasculopathy (PCV), using the Japanese Study Group guidelines [19]. Similarly, 421 healthy participants from the Singapore Chinese Eye Study without AMD nor carrying risk factors such as hypertension, hyperopic refraction and chronic kidney disease, were recruited as control subjects [17, 20]. Throat swabs were stored immediately at -80°C upon collection until deoxyribonucleic acid (DNA) template extraction [21].

### Nucleic acid extraction, 16S rRNA amplification and DNA sequencing

Extraction of total DNA from throat swabs was performed via a combination of mechanical and chemical lysis using FastPrep Instrument (MP Biomedicals) and Qiagen DNeasy EZ1 Advanced XL system (Qiagen). Extraction of samples was conducted without differentiating between Case/Control statuses. An approximate 750bp region (encompassing V3-V6) on prokaryotic 16S rRNA was amplified from total DNA as described previously [22,23]. In summary, we purified DNA amplicons from 35 cycles of PCR using Qiagen Hotstar (Germany), which yielded approximately 1μg of nucleic acids. Amplicons were sheared and constructed into sequencing libraries using GeneRead DNA Library I Core Kit (Qiagen) according to the manufacturer's protocol. DNA libraries were multiplexed using Illumina 48-plex barcodes and 76bp paired-end sequenced on the Illumina HiSeq 2500. After demultiplexing (with the Illumina bcl2fastq 2.17.1.14 software) and removal of reads that failed Illumina’s purity filters (PF = 0), reads were converted to FASTQ format. Reads were then trimmed by removal of trailing bases with quality score ≤ 2. Read pairs containing reads shorter than 60bp were also removed [24].

### Reconstruction and classification of 16S rRNA amplicon sequences

To reconstruct the original amplicon (V3-V6) sequences, we followed the workflow described previously [22,23], which is largely based on EMIRGE [24], an expectation maximization method not only reconstructs amplicons but also provides estimates of relative taxon abundances. In brief, quality-trimmed reads (see above) were input into EMIRGE (GIT version 98787b5). EMIRGE performs template-guided “assembly” based on a modified SILVA SSU (version 102) database that contains sequences between 1200 – 1900bp, using an expectation-maximization algorithm to iterate, align, and assign reads to candidate 16S sequences [24]. This iterative mapping of paired-end reads also prevents chimeric sequences from mapping. The EMIRGE-based reconstruction methodology has been evaluated and benchmarked against modQIIME and RTAX, and was found to be comparatively more robust and to produce highly concordant estimates of taxonomic abundance [22], thus clearly demonstrates its utility for profiling microbiomes in a precise, high-resolution manner.

To reduce computational requirements and runtime, we applied EMIRGE to the top (in terms of average quality) 500,000 reads in each sample [22]. We found this number to be robust enough to accurately reflect the 16S composition in each sample (S1 Fig and S1 Table.) [22]. De facto outputs indicate OTU abundance; where sequences with relative abundance below 0.1% were removed for data quality control.

EMIRGE-reconstructed sequences were trimmed to the primer-amplified section and searched using BLAST against the chimera-checked Greengenes 16S rRNA database (current_GREENGENES_gg16S_unaligned.fasta) [25]. BLAST hits were sorted by (in consecutive order) smallest E-value, highest bit score, highest percent identity, and longest alignment length; only the top hit after this sorting was used for classification. Minimum percent identity levels were set at 80%, 90%, and 95% for the phylum, family, and genus levels respectively; hits below these percentages were dropped and not considered for classification purposes.

### Characterization of microbial community composition

EMIRGE assigns abundance estimates to reconstructed sequences, allowing us to directly use these results to characterize community composition at various taxonomic levels. A relative abundance profile of OTUs was generated for each sample, excluding those that failed to meet the minimum percent identity levels after BLAST (laid out above).

Throat microbiome profiles were successfully obtained (i.e. sufficient 16S PCR product for library building, and >500,000 high quality reads such that EMIRGE reconstruction succeeded) from 245 out of 311 AMD-positive throat swabs, and from 386 out of 421 control throat swabs. Out of the 245 cases, 80 were classified as early-stage AMD and 165 as late-stage AMD (Table 1). Smoking status was available for 126/245 cases and 386/386 controls; only these microbiomes were used for the analysis of the impact of smoking on the pharyngeal microbiome.

**Table 1:** Clinical parameters of study subjects.

	**Cases**	**Controls**	**Total**
**No. of individuals with swabs**	311	421	732
**No. of sequenced individuals w/ clinical data**	245	386	631
** **			
	**Mean ± Standard Deviation (Range) or n (%)**		
**Age**	**Case**	**Control**	**Chi Square (p-value)**
**<60**	41	196	1.58 x 10^−17^
**>60**	204	190	
**Mean age** ± **Standard Deviation**	67.46 ± 8.57 (32–85.26)	60.76 ± 9.95 (45.82–84.39)	
** **			
**Gender**			
**Female**	88	197	2.76 x 10^−4^
**Male**	157	189	
** **			
**Smoking Status**			
**Current or Previous smoking**	48	94	4 x 10^−3^
**Non-smoking**	78	292	
** **			
**Late AMD**			
**Geographic Atrophy (GA)**	7		
**Polypoidal Choroidal Vasculopathy (PCV)**	80		
**Typical Neovascular AMD (tAMD)**	71		
**Mixed Atrophy**	7		
**Early AMD**	80		
**Control**		386	

### Data visualization and statistical analyses

Abundance estimates from EMIRGE were converted to relative abundances at the genus level for each sample. Clinical data, OTU relative abundance tables, Shannon’s and Simpson’s α-diversity indices were processed and calculated using customized R script in R version 3.3.2. OTU relative abundance tables were input for Principal Component Analyses (PCA). The R package “DESeq2” was used to determine differential abundance for case/control conditions (in all samples), and early/late AMD conditions (in case samples). Age and gender were included as covariates. Only taxa found to be significant (P<0.05) were reported.

Generalised Linear Mixed Model (GLMM) was performed to determine genera with significantly different relative abundances between conditions. P-values were corrected for multiple testing using Benjamini-Hochberg correction.

We used “SVA” R package to examine batch effects across samples. Additionally, Principal Coordinate Analyses (PCoA) and Guided Principal Component Analyses (gPCA) [26] were performed to visually inspect for batch effects using “stats” R package.

### Quantitative-PCR (qPCR) of total bacteria and selected microbial genera

Absolute gene counts of total *Prevotella*, *Gemella* and *Streptococcus* were determined with quantitative PCR (Q-PCR). Twenty samples were randomly selected from among the AMD case population for 16S qPCR gene copy determination; this was matched by an equal number of randomly selected control samples. To quantify total bacteria, primers were selected that amplify a uniform 16S region in these genera (S2 Table) [27]. *Prevotella* and *Streptococcus* primers were sourced from Matsuki et al. and Picard et al. respectively [28, 29], while *Gemella* primers were established in-house via multiple sequence alignments of *Gemella* 16S sequences obtained from the NCBI *nr* repository. Primers were tested to ensure specificity to corresponding genera.

Reactions were carried out in 384-well qPCR plates with duplicate 10 μL reaction volumes containing the KAPA SYBR FAST qPCR Master Mix (2X) for LightCycler 480 (Sigma-Aldrich, Inc.), the primers (15 μM) and 2.0 μL of sample DNA. The LightCycler® 480 Instrument II (Roche) was used for amplification and detection with the following thermocycling parameters: 1 cycle of 95°C for 20s followed by amplification at 95°C for 15s, 60°C for 20s for 40 cycles and 1 cycle of 95°C for 15s, 60°C for 15s and 95°C for 15s with readings collected at final step for melting curve analysis. Ct values were recalculated for log copy number/ul DNA from raw data exported into Excel.

Calibration standards for converting Ct values to bacterial 16S rRNA gene copy numbers were generated as follows. Double-stranded DNA oligomers were synthesised (gBLOCK, Integrated DNA Technologies, Inc.) to span respective regions covered by0020corresponding *Prevotella/Gemella/Streptococcus* forward and reverse primers. A 10 ng/μL stock solution was prepared from the lyophilised oligomers as per manufacturer’s instruction. Copy number/μL of stock solution were determined from calculations (DNA concentration / (fragment length x weight of base pair)) and serially diluted to 6 standards with final concentrations spanning 1.0 x 10^3^ to 1.0 x 10^8^ copy numbers. A plot of Ct versus log10(copy number) produced linear calibration curves with typical R^2^ values of 0.99.

Copy numbers for each genera were expressed as percentage of total microbial copy number. Pearson’s correlation coefficient between qPCR and 16S sequencing techniques was determined using Microsoft Excel 2013.

## Results

### Pharyngeal microbiome structure in AMD cases and controls

We successfully obtained bacterial 16S rRNA sequence data from 631 out of 732 individual throat swabs. Of these, 245 were derived from AMD cases, and 386 from controls. Clinical parameters, including age, gender, smoking status, and disease stage are shown in Table 1. With the exception of disease-type variable for AMD cases, pairwise statistical comparisons (PERMANOVA) of microbial community abundance among case and control samples revealed no obvious stratification of pharyngeal microbiome profiles by gender and age variables (S3 Table). No significant surrogate variables were identified from “SVA” analysis (data not shown). Visual inspection for batch effects using Principal Coordinate Analysis (PCoA), similarly indicated an absence of batch effects among samples (S2 Fig). Finally, application of Guided Principal Components Analysis (gPCA) revealed that proportion of variance due to batch effects was not significantly greater than would be expected (p<0.05) (S3 Fig).

**Fig 1:**
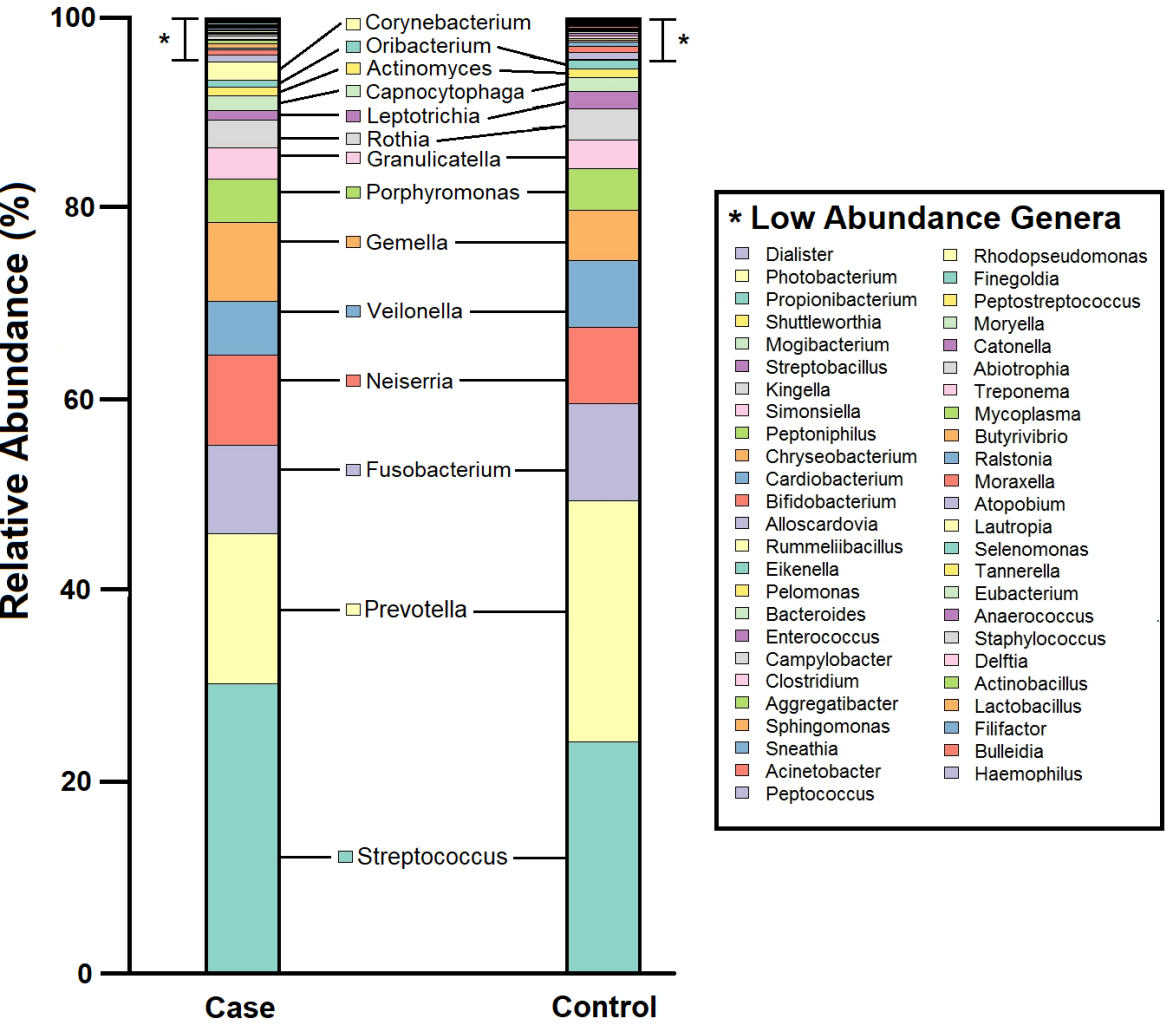
Pharyngeal microbiome profile of the study population, at genus level. Cumulative relative abundance of each of the 63 bacterial genera in case and control samples. Microbial community members with relative abundance <0.1% are listed as Low Abundance Genera (indicated by “*”).

In total, nine phyla and 63 genera were detected across all 631 subjects. The mean number of identified genera were 17 (*SD* = 6) and 18 (*SD* = 5) in 245 cases and 386 controls respectively. This reflects some degree of similarity in the number of identified genera from samples collected from a specific anatomical niche, albeit with varied disease status. Consistent with previous reports [1], two dominant phyla—Firmicutes and Bacteroidetes—accounted for more than half of all OTUs. Members of the microbial community in case and control samples were similar, however, relative abundances of each member differed between case (n = 245) and control (n = 386) groups (Fig 1). In terms of genera prevalence among study population, *Streptococcus*, *Prevotella*, *Veilonella* and *Gemella* were most prevalent in both case (n = 245) and control (n = 386) samples, being detected in >85% of all subjects (S4 Fig). Shannon and Simpson diversity indices did not differ significantly between cases and controls (p < 0.05, Mann-Whitney U test) (S5 Fig).

Principal Components Analysis (PCA) revealed the lack of clustering across conditions, implying that maximum abundance variances are insufficient to distinguish between conditions. It also suggests that genera relative abundances between conditions are subtle (S3 Fig).

### Association of specific microbial genera with AMD

**Fig 2:**
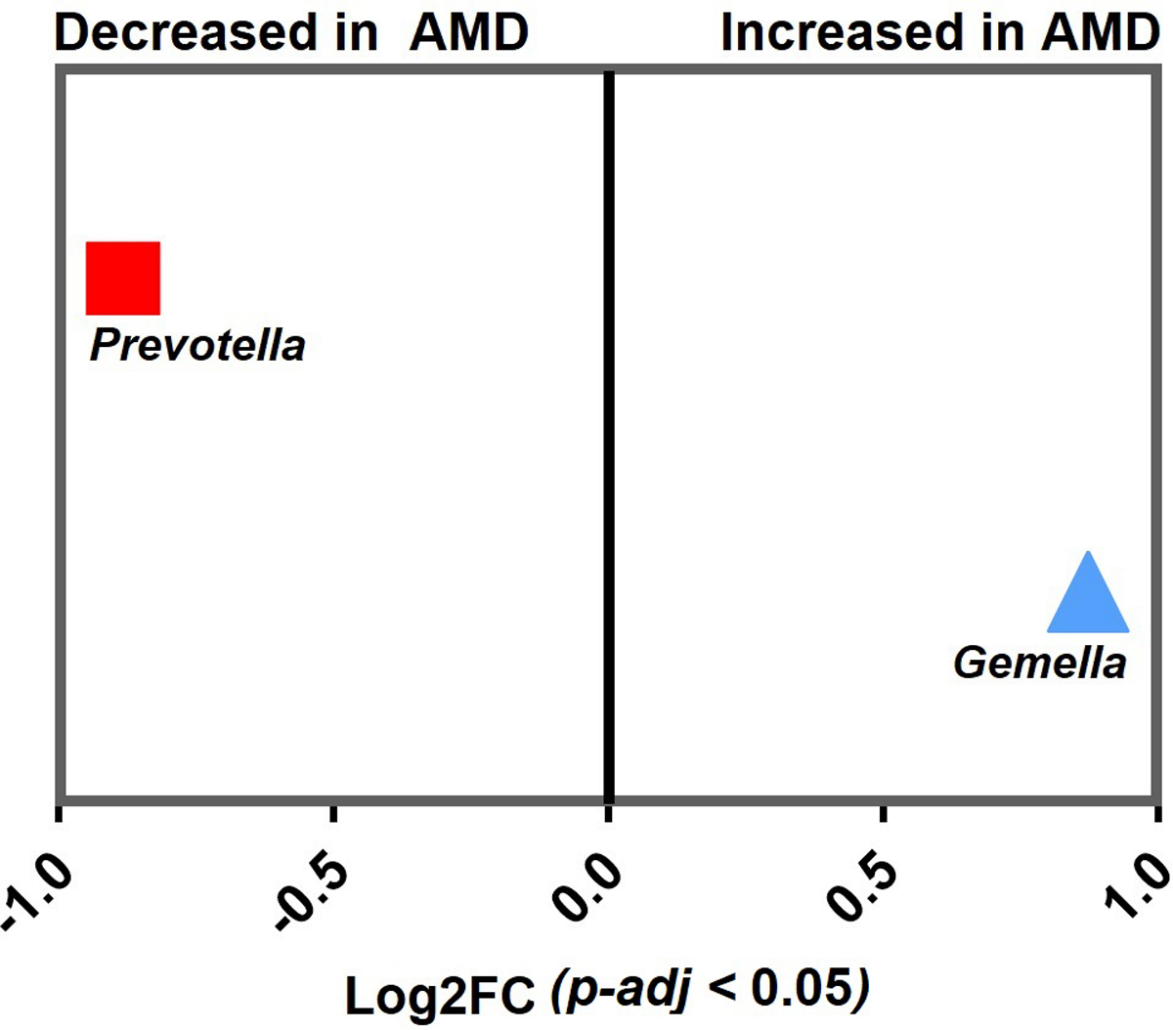
DESeq2 differential abundance analysis expressed as Log2FC comparison of AMD-positive samples and control samples. Negative fold change scores (log2) indicate genera with decreased abundance in AMD-positive samples, and positive fold change scores indicate genera with increased abundance in AMD-positive samples. Each point represents a genus. Genera detected to have significant difference in abundance (*Adj-p* < 0.05) are shown.

**Fig 3:**
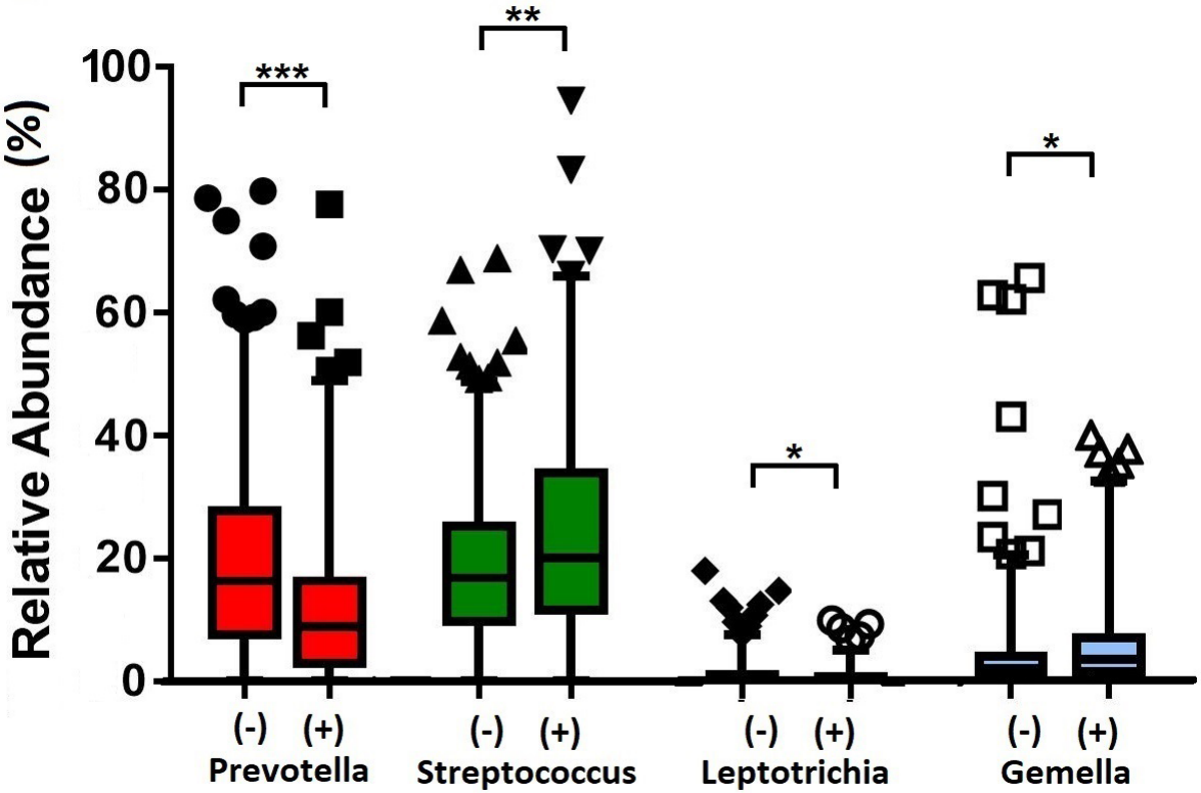
Relative abundances of significant genera between 245 case and 386 control samples. AMD-positive and control samples are denoted by (+) and (-) respectively. Statistical significance is indicated by (*Adj-p < 0.05), (**Adj-p < 0.005) or (***Adj-p < 0.0005). Mean relative abundances, standard deviations and P-values are presented in S4 Table.

245 case and 386 control samples were used for DESeq2 to evaluate differential abundance of bacterial genera. DESeq2 analyses found the case samples to be enriched for *Gemella* (*Adj-p* = 3.15 x 10^−5^), while *Prevotella* had reduced relative abundance (*Adj-p* = 1.89 x 10^−5^) (Fig 2). While, in the comparisons between early versus late AMD cases, no genera was detected to be significantly different in relative abundance among the groups. Likewise was in the smoking status comparisons (*Adj-p* < 0.05). Using Generalised Linear Mixed Model analysis, we observed the robust associations of *Prevotella* and *Gemella* with case/control status, as similarly shown in the DESeq2 analysis. In addition, we detected *Streptococcus* and *Leptotrichia* to associate with case/control status as well (Fig 3 and S4 Table). *Streptococcus* and *Prevotella*, the two most abundant genera, showed an opposite trend: *Streptococcus* was present at a significantly higher abundance in cases than in controls (mean relative abundance of 23.4% vs 18.6%; *Adj-p* = 0.002), while *Prevotella* was present at a significantly lower abundance (12.7% vs 19.3%; *Adj-p* = 6.95 x 10^−5^). In addition, *Gemella* was significantly more abundant in cases than in controls (6.0% vs 4.0%; *Adj-p* = 0.007). *Leptotrichia* which were present at low relative abundance (<1%), also differed significantly (*Adj-p* = 0.007) between cases and controls (Fig 3 and S4 Table). Analysing the >60 years subset revealed only *Prevotella*, *Leptotrichia* and *Streptococcus* to be significantly associated (*Streptococcus Adj-p* = 1.85 x 10^−5^; *Leptotrichia Adj-p* = 0.005; *Streptococcus Adj-p* = 0.035) among cases and controls (S5 Table).

**Fig 4:**
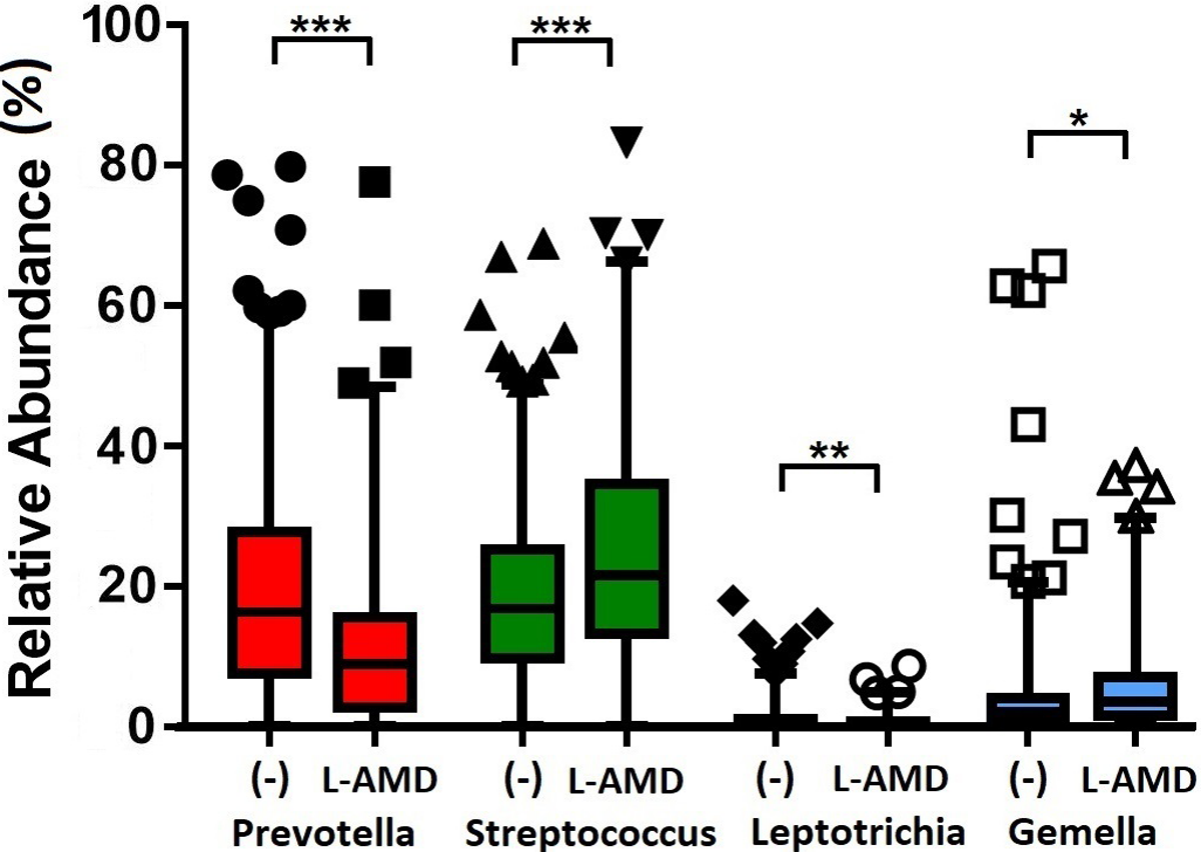
Relative abundances of significant genera between 386 controls and 165 individuals with late AMD. (-) and L-AMD denote controls and late AMD samples respectively. Statistical significance is indicated by (*Adj-p < 0.05), (**Adj-p < 0.005) or (***Adj-p < 0.0005). Mean relative abundances, standard deviations and P-values are presented in S6 Table.

Stratifying the AMD cases by disease into early and late stages, *Prevotella* and *Leptotrichia* relative abundance was significantly lower in late AMD samples than controls (Fig 4 and S6 Table). Conversely, late AMD samples were revealed to have greater relative abundances of *Streptococcus* and *Gemella* (*Streptococcus Adj-p =* 1.19 x 10^−4^; *Gemella Adj-p =* 0.028) (Fig 4). Comparing early AMD samples to controls did not reveal significant differences in genera relative abundances between both conditions. Further analysis to compare microbiomes between subgroups of late AMD disease (as described in Table 1) was under powered by reduced numbers of individuals, but remains an area of future interest.

Sampling rarely yields individual microbes in isolation; instead, microbes exist in communities together with other bacterial genera. To understand bacterial communities associated with *Prevotella* and *Gemella*, we identified subjects with high relative abundance of each genus, defined as relative abundance greater than two standard deviations above the population mean. Comparing the microbiota of these high abundance groups with the rest of the study population revealed distinct subsets of bacterial genera that are associated with each genus (Tables 2 and 3).

**Table 2:** Microbial genera significantly associated with high *Prevotella* relative abundance.

	Mean relative abundance ± Standard Deviation		
	Rest (n = 604)	High Prevotella (n = 27)	Adj. P-value
**Streptococcus**	0.204 ± 0.144	0.068 ± 0.069	6.11 x 10^−5^
**Porphyromonas**	0.035 ± 0.045	0.003 ± 0.006	0.005
**Granulicatella**	0.025 ± 0.026	0.007 ± 0.01	0.008
**Gemella**	0.05 ± 0.073	0.005 ± 0.006	0.042

**Table 3:** Microbial genera significantly associated with high *Gemella* relative abundance.

	Mean relative abundance ± Standard Deviation		
	Rest (n = 610)	High Gemella (n = 21)	Adj. P-value
**Aggregatibacter**	2.1 x 10–4 ± 7 x 10–4	0.001 ± 0.003	1.9 x 10^−4^
**Prevotella**	0.182 ± 0.156	0.042 ± 0.055	0.001
**Filifactor**	0.002 ± 0.009	0.016 ± 0.057	0.002
**Haemophilus**	0.005 ± 0.0122	0.016 ± 0.03	0.004
**Veillonella**	0.054 ± 0.053	0.01 ± 0.019	0.006

High *Prevotella* abundance was uniquely associated with reduced *Gemella*, *Granulicatella*, *Porphyromonas and Streptococcus* abundance, and high *Gemella* abundance was uniquely associated with higher *Filifactor*, *Haemophilus and Aggregatibacter* abundances. Using PCA to map samples harbouring either high *Prevotella* or high *Gemella* abundances, a distinct cluster of 27 samples was observed with high *Prevotella* abundance, of which six were case samples (S6 Fig).

### Quantification-PCR (qPCR) validation of selected genera

**Fig 5:**
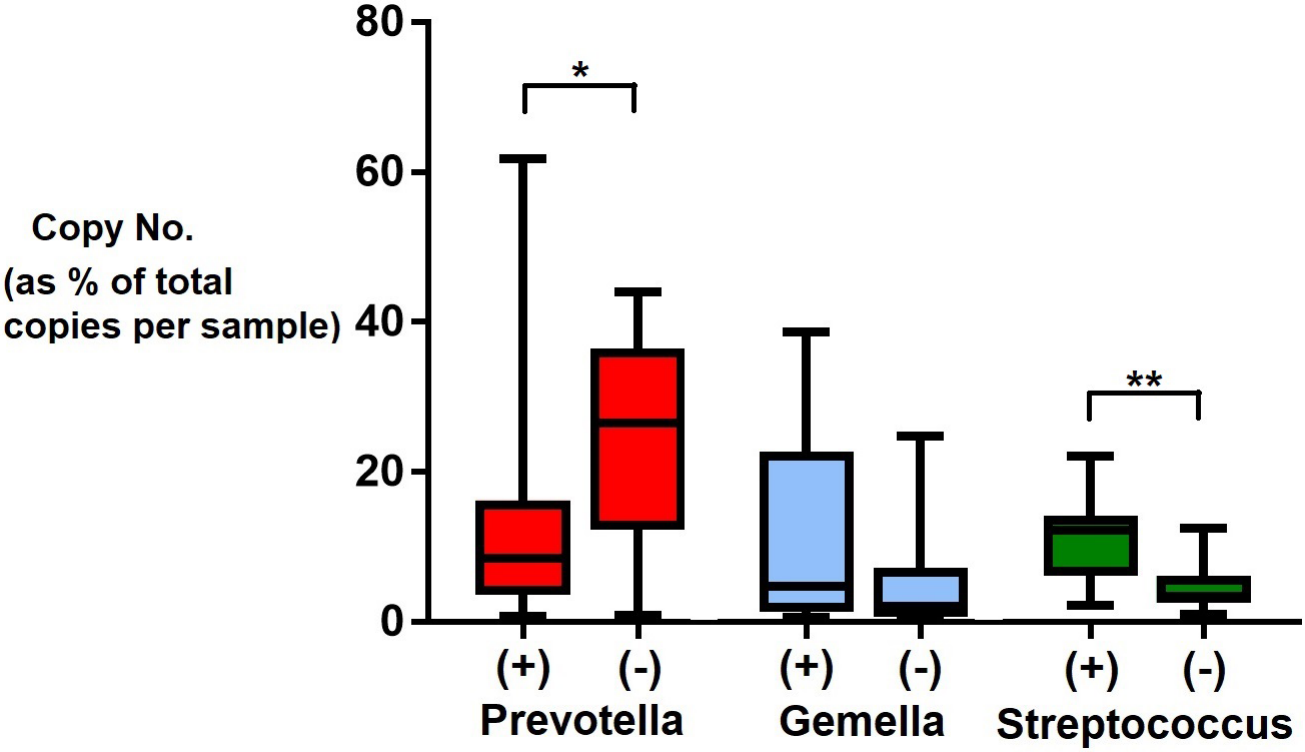
Microbial loads from qPCR. qPCR-derived copy numbers of each genera were expressed as a percentage of total microbial copy number within each sample. Twenty samples were randomly selected for each disease status. Statistical significance is indicated by (*P < 0.01) or (**P < 0.001) as determined by Mann-Whitney U test.

To test the ability of our 16S sequencing to detect differences in abundance, we utilised qPCR assays as a validation platform to quantify microbial loads of three highly prevalent genera exhibiting narrow difference in mean abundance among controls and cases. Pearson’s correlation showed both techniques were positively correlated to some extent (R = 0.322). Microbial loads reported by qPCR demonstrated a similar trend as relative abundance by sequencing for case/control status. Using qPCR, *Streptococcus* was significantly enriched in case samples and *Prevotella* in controls. *Gemella* was increased in case samples, however, this difference was not significant by qPCR (Fig 5). While we found that 16S sequencing and qPCR are consistent in capturing overall microbial loads, the choice of different technology will result in different outputs and data formats. In addition to abundance estimates, 16S sequencing is able to identify members of a microbial community and their respective quantities in relation to other members.

## Discussion

We have comprehensively and accurately described the human pharyngeal microbiome, at genus level in this a large case-control study of 631 AMD subjects, by shotgun sequencing the V3-V6 region of the microbial 16S rRNA gene. Consistent with previous studies [1,30–33], pharyngeal microbiomes in our study population were dominated by relatively few bacterial genera, of which *Streptococcus* and *Prevotella* were most abundant and prevalent. These "core" genera may play roles in maintaining the stability and complexity of individual microbiomes over time, properties thought to be associated with health [1].

Investigating community relative abundances using PCA revealed that the microbial drivers in the pharyngeal community are not vastly different between cases and controls. Rather, they suggest that the pharyngeal community is overall stable in terms of community genera members and their respective relative abundances across both disease and health conditions. Overall, it is interesting to note that pharyngeal community relative abundances in early AMD were more similar to healthy controls, while late AMD condition revealed significant dissimilarity in community relative abundance when compared to controls, which potentially suggests some dysbiosis related to disease progression. AMD is a progressive disease, and this observation suggests that alterations in the pharyngeal microbiota becomes more evident as the disease progresses. Current treatment of “wet” AMD is based on suppression of vascular endothelial growth factor (VEGF) with intraocular injections of anti-VEGF agents. While this has significantly improved the outcomes in the management of AMD, this treatment does not reverse damage related to AMD, is invasive, expensive, and incurs significant healthcare resources. Thus, a search for the underlying cause of AMD with strategies to prevent its development and progression remains a key research priority.

*Gemella* and *Prevotella* were detected to have significant differential abundance between case and control conditions. Difference in mean relative abundance of both genera in disease and health conditions also reached statistical significance. This could either suggest that health/disease conditions could have an impact on community structure, or community abundances play a role towards disease propagation. Our findings provide preliminary insight into the composition of pharyngeal microbiota, as well as candidate genera with their abundance alterations in AMD health and disease conditions. Metagenomic sequencing and translational studies can be performed in future to provide more robust evidence to elucidate relevant biological factors leading to this observation.

We were also able to investigate potential roles for pharyngeal microbiome members in the initiation and pathogenesis of AMD. While overall diversity and composition of pharyngeal microbiomes from AMD cases and controls were relatively similar, we observed clear differences in specific bacterial genera between the two groups. *Gemella* and *Prevotella* (also the two most abundant genera), showed opposite trends, with *Gemella* being more abundant in cases and *Prevotella* more so in controls. *Gemella* is a dominant member of the laryngeal mucosal community and has been identified to be present at higher abundances in patients with laryngeal squamous cell carcinoma than healthy subjects [34, 35]. *Prevotella* species are not known to be protective; some such as *Prevotella intermedia* are known to cause periodontal disease [36], and *Prevotella copri* in the gut has also been associated with rheumatoid arthritis [37]. While there is a possibility that the protective effect we observed here for AMD is due to corresponding lower levels of *Gemella* in individuals with high *Prevotella* loads, nevertheless it begets the question of why some community members show differential abundance in health or disease conditions.

To better understand the composition of communities harbouring high *Prevotella* and *Gemella* loads, we looked for community members associated with high *Prevotella* and Gemella. Most associations were negative, i.e. high driver abundance correlated with reduced abundance of the genus in question. This could reflect the fact that the drivers were the most abundant genera (such that other genera are correspondingly low), or alternatively could indicate the existence of competitive mechanisms. Conversely, positive associations (for example, high *Gemella* was associated with high *Haemophilus*, *Filifactor* and *Aggregatibacter*) could indicate cooperative or symbiotic interactions. These patterns suggest that intra-community interactions are likely to affect the overall contribution of the individual microbiota to AMD pathogenesis. PCA clustering revealed high *Prevotella* samples (n = 27) to form an exclusive cluster, suggesting the high *Prevotella* subset to possess unique microbial profile against the rest of the samples. Among this subset were six case samples, indicating that while increased *Prevotella* abundance is associated with control status, it is not the sole scenario. It suggests greater complexity between association of the pharyngeal microbiota and AMD to be beyond the sheer abundance of a single genus.

Host genetic differences between cases and controls may also influence pharyngeal microbiome composition and colonization efficiency. A recent study revealed the rs3006458-T allele (associated with lower abundance of *Aerococcus* and *Micrococcacea* in the nasopharynx) [38] was associated with increased PGLYRP4 expression in mucosal tissues supporting antimicrobial innate immunity [39]. These data suggest that host genotype regulates PGLYRP4 expression on the respiratory mucosa that leads to modulatory effect on bacterial abundance [38]. A number of pathogenic bacteria, including *Neisseria meningitidis*, *Streptococcus pneumoniae*, *Streptococcus pyogenes*, and *Borrelia burgdoferi* encode surface proteins that bind to and recruit CFH, thus mediating evasion of complement-mediated killing [40, 41]. Genetic variants in CFH associated with increased AMD risk have been shown to alter binding to *S*. *pyogenes* surface proteins, and consequently impact complement activation, opsonization, and phagocytosis [42,43]. In addition, individuals carrying the Tyr402His CFH risk variant who also harbored high levels of antibodies against *Chlamydia pneumoniae* showed an increased risk of AMD progression [6]. Our work provides further evidence that these factors play a role in AMD disease and progression.

## Conclusions

Through 16S sequencing of an extensive population cohort, this study has provided accurate representation of the population as well as considerable confidence in a role for the pharyngeal microbiota profile in AMD health and disease conditions. Pharyngeal microbiota members and their relative abundances are highly stable among populations. Subtle differences were identified in relative abundances of *Prevotella*, *Gemella*, *Streptococcus* and *Leptotrichia* in the pharyngeal composition of AMD cases when compared to controls, a phenomenon similarly observed in comparisons between controls and patients with late-stage AMD. Prospective studies investigating pharyngeal microbiomes prior to disease, longitudinal studies tracking microbiomes over the course of disease progression, and analysis of genetic factors influencing microbiome composition will be necessary to fully characterize microbial triggers of AMD.

## Supporting information

S1 Fig Rarefaction curves indicating the number of genera detected with less than <500,000 reconstructed reads in 30 randomly picked samples. Samples are labelled with “PHT” prefix and corresponding sample number.
(DOCX)

S2 Fig Visual inspection for batch effects using Principal Coordinate Analysis (PCoA) based on relative abundance at genus level. Each point represents an individual.
(DOCX)

S3 Fig **Guided Principal Component Analysis (gPCA) based on relative abundance on (A) Gender, (B) Disease status (C) Age and (D) Disease progression factors.** Each point represents an individual. Case samples has a similar community composition similar to that of control samples. Additionally, microbial community composition is highly similar among early/late AMD status.
(DOCX)

S4 Fig Prevalence (percentage of individuals in which the genus was detected) of each microbial genus in case and control samples. (DOCX)

S5 Fig Measures of pharyngeal microbiome community diversity. Shannon and Simpson diversity indices for pharyngeal microbiomes in AMD cases and controls. Each data point represents an individual pharyngeal microbiome; boxes indicate the mean and 25th and 75th percentiles; whiskers indicate interquartile ranges. Shannon and Simpson diversity indices did not differ significantly between cases and controls (p < 0.05, Mann-Whitney U test).
(DOCX)

S6 Fig **PCA plots colored by samples harbouring (A) high *Prevotella* (n = 27) and (B) high *Gemella* (n = 21) relative abundances.** High *Prevotella* samples clustered at the tip of the plot (A).
(DOCX)

S1 Table Comparison of identified genera in 30 samples using 500,000 and <500,000 reconstructed reads. (DOCX)

S2 Table Quantitative-PCR (qPCR) primers and their corresponding amplicon sizes. (DOCX)

S3 Table Gender, age and disease-type Pairwise Statistical Comparisons (PERMANOVA) of microbial community abundance among case and control samples. (DOCX)

S4 Table Association between microbial genera and AMD status. Genera with significantly different relative abundances in case/control conditions are shown.
(DOCX)

S5 Table Association between microbial genera and AMD status in individuals >60 years. Genera with significantly different relative abundances in case/control conditions are shown.
(DOCX)

S6 Table Association between microbial genera with control and Late AMD status. Genera with significantly different relative abundances in control and Late AMD conditions are shown.
(DOCX)
