# Supplementary material for: Human pharyngeal microbiota in age-related macular degeneration
Source: PLoS One. 2018 Aug 8;13(8):e0201768. doi: 10.1371/journal.pone.0201768 (PMC6082546; doi:10.1371/journal.pone.0201768)
Supplement: S1 Fig — Samples are labelled with “PHT” prefix and corresponding sample number. (DOCX) [file pone.0201768.s001.docx]

**Supplemental Material**


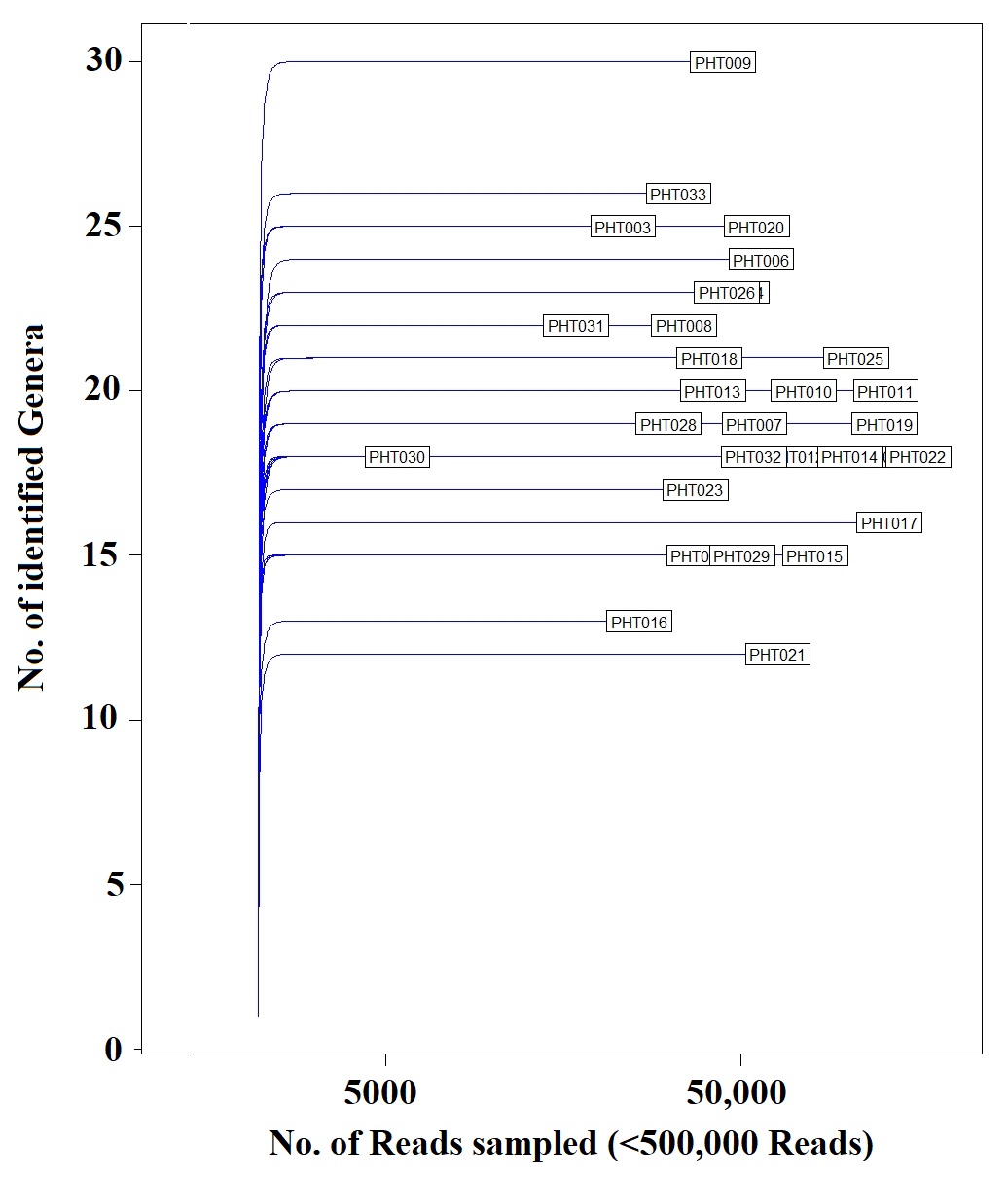


**Supplementary Figure 1.** Rarefaction curves indicating the number of genera detected with less than <500,000 reconstructed reads in 30 randomly picked samples. Samples are labelled with “PHT” prefix and corresponding sample number.
