# Supplementary material for: Human pharyngeal microbiota in age-related macular degeneration
Source: PLoS One. 2018 Aug 8;13(8):e0201768. doi: 10.1371/journal.pone.0201768 (PMC6082546; doi:10.1371/journal.pone.0201768)
Supplement: S2 Fig — Each point represents an individual. (DOCX) [file pone.0201768.s005.docx]

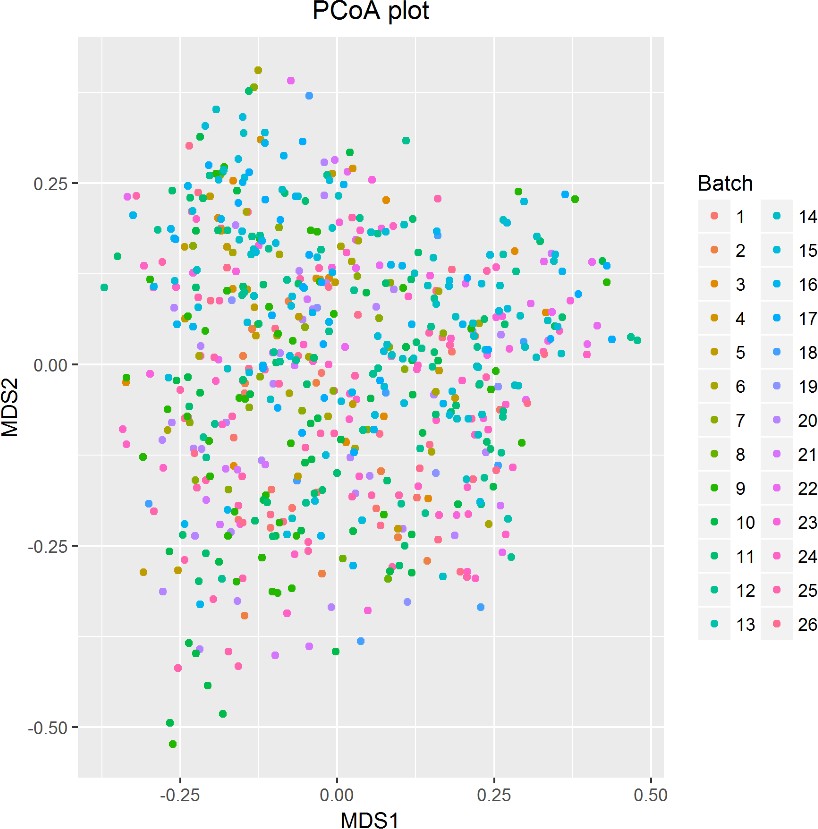
1 **Supplemental Material**

2

1. **Supplementary Figure 2:** Visual inspection for batch effects using Principal Coordinate
2. Analysis (PCoA) based on relative abundance at genus level. Each point represents an
3. individual.
