# Supplementary material for: Human pharyngeal microbiota in age-related macular degeneration
Source: PLoS One. 2018 Aug 8;13(8):e0201768. doi: 10.1371/journal.pone.0201768 (PMC6082546; doi:10.1371/journal.pone.0201768)
Supplement: S3 Fig — Guided Principal Component Analysis (gPCA) based on relative abundance on (A) Gender, (B) Disease status (C) Age and (D) Disease progression factors. Each point represents an individual. Case samples has a similar community composition similar to that of control samples. Additionally, microbial community composition is highly similar among early/late AMD status. (DOCX) [file pone.0201768.s006.docx]

**Supplemental Material**

**
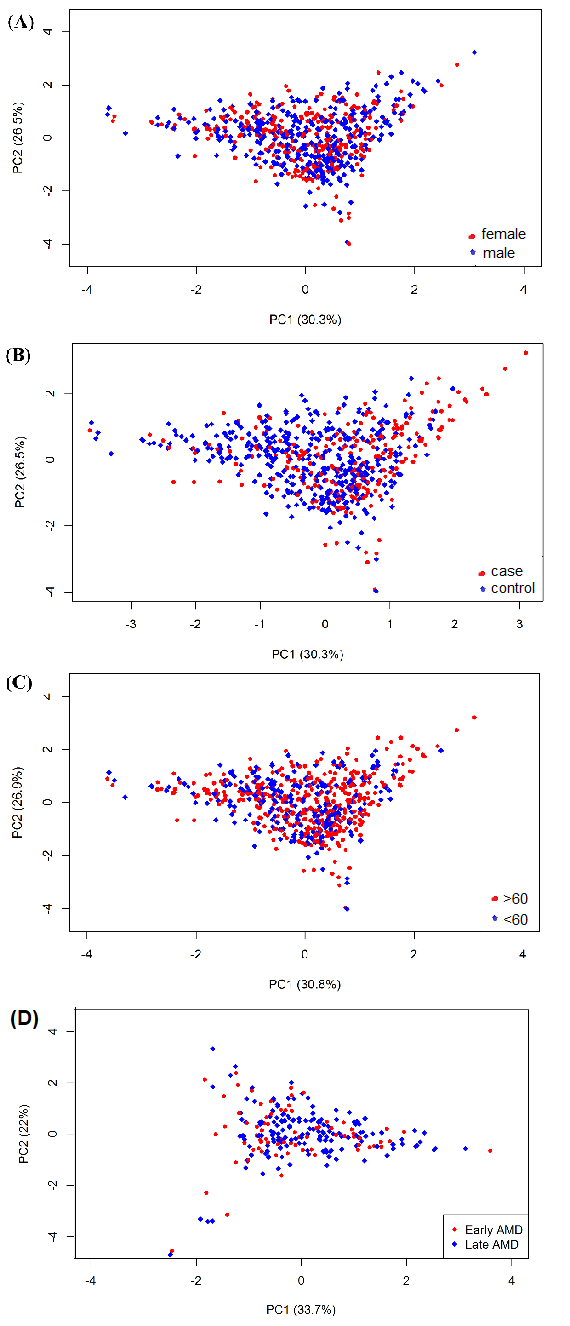
**

**Supplementary Figure 3:** Guided Principal Component Analysis (gPCA) based on relative

abundance on (A) Gender, (B) Disease status (C) Age and (D) Disease progression factors. Each point represents an individual. Case samples have a similar community composition similar to that of control samples. Additionally, microbial community composition is highly similar among early/late AMD status.
