# Supplementary material for: Human pharyngeal microbiota in age-related macular degeneration
Source: PLoS One. 2018 Aug 8;13(8):e0201768. doi: 10.1371/journal.pone.0201768 (PMC6082546; doi:10.1371/journal.pone.0201768)
Supplement: S4 Fig — (DOCX) [file pone.0201768.s010.docx]

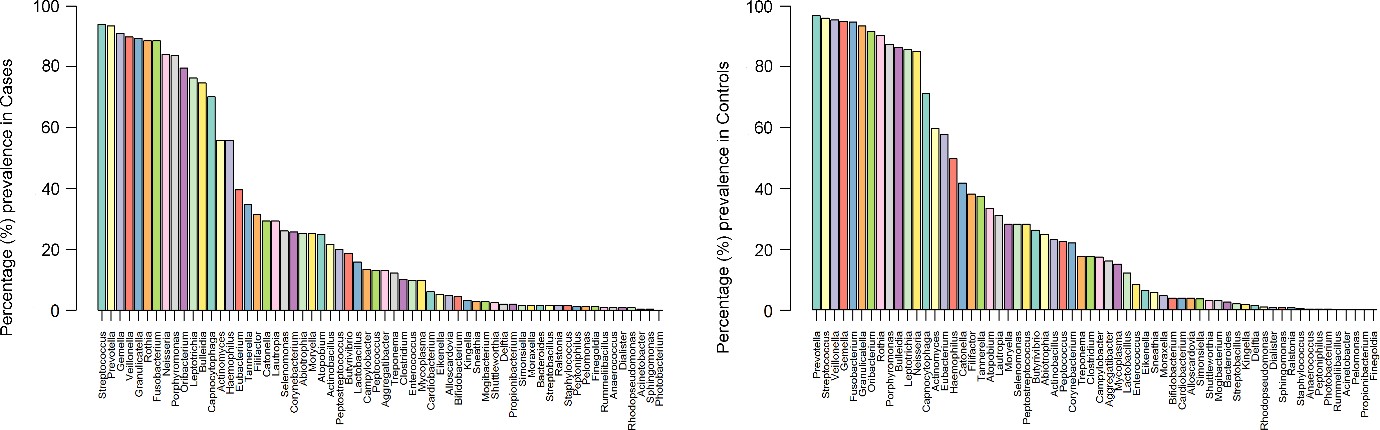
1 **Supplemental Material**

2

1. **Supplementary Figure 4.** Prevalence (percentage of individuals in which the genus was
2. detected) of each microbial genus in case and control samples.
