# Supplementary material for: Human pharyngeal microbiota in age-related macular degeneration
Source: PLoS One. 2018 Aug 8;13(8):e0201768. doi: 10.1371/journal.pone.0201768 (PMC6082546; doi:10.1371/journal.pone.0201768)
Supplement: S5 Fig — Shannon and Simpson diversity indices for pharyngeal microbiomes in AMD cases and controls. Each data point represents an individual pharyngeal microbiome; boxes indicate the mean and 25th and 75th percentiles; whiskers indicate interquartile ranges. Shannon and Simpson diversity indices did not differ significantly between cases and controls (p < 0.05, Mann-Whitney U test). (DOCX) [file pone.0201768.s011.docx]

#
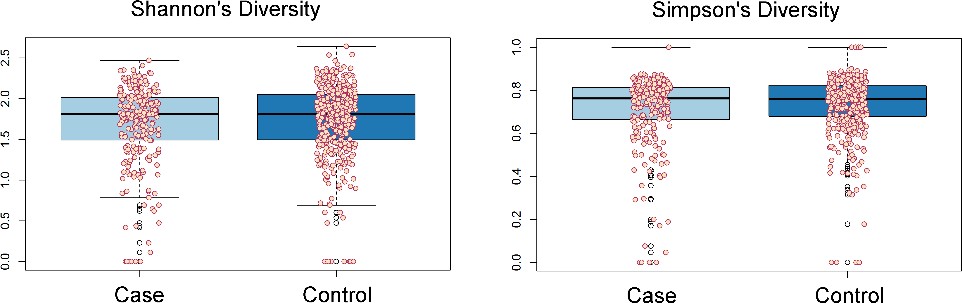
1 Supplemental Material

2

# Supplementary Figure 5. Measures of pharyngeal microbiome community diversity.

1. Shannon and Simpson diversity indices for pharyngeal microbiomes in AMD cases and
2. controls. Each data point represents an individual pharyngeal microbiome; boxes indicate the
3. mean and 25th and 75th percentiles; whiskers indicate interquartile ranges. Shannon and
4. Simpson diversity indices did not differ significantly between cases and controls (p < 0.05,
5. Mann-Whitney U test).
