# Supplementary material for: Human pharyngeal microbiota in age-related macular degeneration
Source: PLoS One. 2018 Aug 8;13(8):e0201768. doi: 10.1371/journal.pone.0201768 (PMC6082546; doi:10.1371/journal.pone.0201768)
Supplement: S6 Fig — PCA plots colored by samples harbouring (A) high Prevotella (n = 27) and (B) high Gemella (n = 21) relative abundances. High Prevotella samples clustered at the tip of the plot (A). (DOCX) [file pone.0201768.s012.docx]

**Supplemental Material**


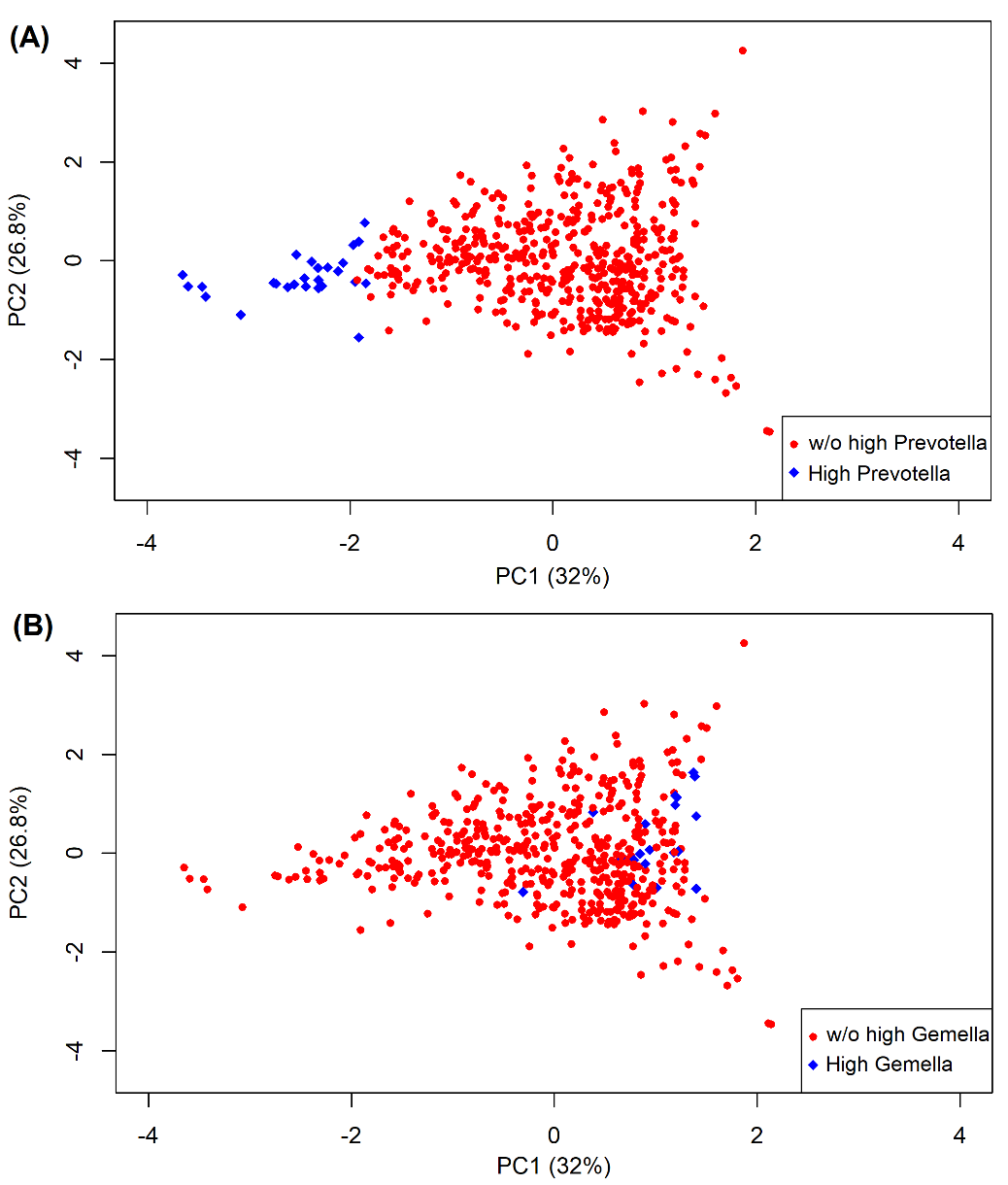


**Supplementary Figure 6.** PCA plots colored by samples harbouring (A) high *Prevotella* (n=27) and (B) high *Gemella* (n=21) relative abundances. High *Prevotella* samples clustered at the tip of the plot (A).
