# Supplementary material for: Human pharyngeal microbiota in age-related macular degeneration
Source: PLoS One. 2018 Aug 8;13(8):e0201768. doi: 10.1371/journal.pone.0201768 (PMC6082546; doi:10.1371/journal.pone.0201768)
Supplement: S1 Table — (DOCX) [file pone.0201768.s002.docx]

**Supplemental Material**

| **Sample** | **No. of genera using 500,000 reconstructed reads** | **No. of genera using <500,000 reconstructed reads** |
| --- | --- | --- |
| PHT009 | 30 | 30 |
| PHT033 | 26 | 26 |
| PHT003 | 25 | 25 |
| PHT020 | 25 | 25 |
| PHT006 | 24 | 24 |
| PHT024 | 23 | 23 |
| PHT026 | 23 | 23 |
| PHT031 | 22 | 22 |
| PHT008 | 22 | 22 |
| PHT018 | 21 | 21 |
| PHT025 | 21 | 21 |
| PHT013 | 20 | 20 |
| PHT010 | 20 | 20 |
| PHT011 | 20 | 20 |
| PHT028 | 19 | 19 |
| PHT007 | 19 | 19 |
| PHT019 | 19 | 19 |
| PHT036 | 18 | 18 |
| PHT032 | 18 | 18 |
| PHT012 | 18 | 18 |
| PHT014 | 18 | 18 |
| PHT022 | 18 | 18 |
| PHT030 | 18 | 18 |
| PHT023 | 17 | 17 |
| PHT017 | 16 | 16 |
| PHT027 | 15 | 15 |
| PHT029 | 15 | 15 |
| PHT015 | 15 | 15 |
| PHT016 | 13 | 13 |
| PHT021 | 12 | 12 |

**Supplementary** **Table 1**. Comparison of identified genera in 30 samples using 500,000 and <500,000 reconstructed reads (Figure 1).
