# Supplementary material for: Human pharyngeal microbiota in age-related macular degeneration
Source: PLoS One. 2018 Aug 8;13(8):e0201768. doi: 10.1371/journal.pone.0201768 (PMC6082546; doi:10.1371/journal.pone.0201768)
Supplement: S2 Table — (DOCX) [file pone.0201768.s003.docx]

**Supplemental Material**

**Supplementary Table 2.** Quantitative-PCR **(**qPCR) primers and their corresponding amplicon sizes.

| **Amplicon target** | **Primer Label** | **Sequence (5' - 3')** | **Amplicon Length (bp)** | **References** |
| --- | --- | --- | --- | --- |
| **Total Bacteria** | 338F | ACT CCT ACG GGA GGC AGC | 219 | Chua et al, 2017 |
|  | 533R | TTA CCG CGG CTG CTG GCA C |  |  |
| **Streptococcus spp.** | Str1-F | GTA CAG TTG CTT CAG GAC GTA TC | 208 | Picard et al, 2004 |
|  | Str2-R | ACG TTC GAT TTC ATC ACG TTG |  |  |
| **Prevotella spp.** | bg-Prevo-F | CAC RGT AAA CGATGG ATG CC | 523 | Matsuki et al, 2004 |
|  | bg-Prevo-R | GGT CGG GTT GCA GAC C |  |  |
| **Gemella spp.** | Gemella-F_geneious design | TCA ACC GTG GAG GGT CAT TG | 531 | Designed In-house |
|  | Gemella-R_geneious design | CCC CAT CTT ACT GCT GGC AA |  |  |
