# Supplementary material for: Human pharyngeal microbiota in age-related macular degeneration
Source: PLoS One. 2018 Aug 8;13(8):e0201768. doi: 10.1371/journal.pone.0201768 (PMC6082546; doi:10.1371/journal.pone.0201768)
Supplement: S3 Table — (DOCX) [file pone.0201768.s004.docx]

1. **Supplemental Material**
2. **Supplementary Table 3.** Gender, age and disease-type Pairwise Statistical Comparisons
3. (PERMANOVA) of microbial community abundance among case and control samples.

|  | **R2** | **Pr (>F)** |
| --- | --- | --- |
| **Gender** | 0.001 | 0.751 |
| **Age** | 0.004 | 0.055 |
| **Disease type (GA,PCV,tAMD,Mixed Atrophy, Early)** | 0.021 | 0.005 |

4
