# Supplementary material for: Human pharyngeal microbiota in age-related macular degeneration
Source: PLoS One. 2018 Aug 8;13(8):e0201768. doi: 10.1371/journal.pone.0201768 (PMC6082546; doi:10.1371/journal.pone.0201768)
Supplement: S4 Table — Genera with significantly different relative abundances in case/control conditions are shown. (DOCX) [file pone.0201768.s007.docx]

**Supplemental Material**

**Supplementary Table 4: Association between microbial genera and AMD status.** Genera with significantly different relative abundances in case/control conditions are shown.

|  | **Mean relative abundance ± Standard Deviation** | |  |
| --- | --- | --- | --- |
|  | **Control (n=386)** | **Case (n=245)** | **Adj. p-value** |
| **Prevotella** | 0.193 ± 0.157 | 0.127 ± 0.133 | 6.95 x 10^-5^ |
| **Streptococcus** | 0.186 ± 0.129 | 0.234 ± 0.173 | 0.002 |
| **Leptotrichia** | 0.014 ± 0.023 | 0.008 ± 0.015 | 0.007 |
| **Gemella** | 0.04 ± 0.071 | 0.06 ± 0.078 | 0.007 |
