# Supplementary material for: Human pharyngeal microbiota in age-related macular degeneration
Source: PLoS One. 2018 Aug 8;13(8):e0201768. doi: 10.1371/journal.pone.0201768 (PMC6082546; doi:10.1371/journal.pone.0201768)
Supplement: S5 Table — Genera with significantly different relative abundances in case/control conditions are shown. (DOCX) [file pone.0201768.s008.docx]

**Supplemental Material**

**Supplementary Table 5.** Association between microbial genera and AMD status in individuals >60 years. Genera with significantly different relative abundances in case/control conditions are shown.

|  | **Mean relative abundance ± Standard Deviation** | |  |
| --- | --- | --- | --- |
|  | **Control (n=180)** | **Case (n=192)** | **Adj. p-value** |
| **Prevotella** | 0.2 ± 0.157 | 0.123 ± 0.129 | 1.85 x 10^-5^ |
| **Leptotrichia** | 0.016 ± 0.026 | 0.008 ± 0.015 | 0.005 |
| **Streptococcus** | 0.189 ± 0.132 | 0.236 ± 0.175 | 0.035 |
