# Supplementary material for: Human pharyngeal microbiota in age-related macular degeneration
Source: PLoS One. 2018 Aug 8;13(8):e0201768. doi: 10.1371/journal.pone.0201768 (PMC6082546; doi:10.1371/journal.pone.0201768)
Supplement: S6 Table — Genera with significantly different relative abundances in control and Late AMD conditions are shown. (DOCX) [file pone.0201768.s009.docx]

**Supplemental Material**

**Supplementary Table 6: Association between microbial genera with Control and Late AMD status.** Genera with significantly different relative abundances in control and Late AMD conditions are shown.

|  | **Mean relative abundance ± Standard Deviation** | |  |
| --- | --- | --- | --- |
|  | **Control (n=386)** | **Late AMD (n=165)** | **Adj. p-value** |
| **Prevotella** | 0.193 ± 0.157 | 0.123 ± 0.131 | 1.19 x 10^-4^ |
| **Streptococcus** | 0.186 ± 0.129 | 0.25 ± 0.173 | 1.19 x 10^-4^ |
| **Leptotrichia** | 0.014 ± 0.023 | 0.007 ± 0.012 | 0.003 |
| **Gemella** | 0.04 ± 0.071 | 0.06 ± 0.072 | 0.028 |
